# UNDERSTANDING CHANGES IN PUBLIC TRANSIT BEHAVIOR AND ITS LINKS TO PHYSICAL ACTIVITY: FINDINGS FROM THE NHATS

**DOI:** 10.1093/geroni/igae098.1198

**Published:** 2024-12-31

**Authors:** Erica Twardzik, Jennifer Schrack

**Affiliations:** University of Michigan, Ann Arbor, Maryland, United States; Johns Hopkins Bloomberg School of Public Health, Baltimore, Maryland, United States

## Abstract

Public transit use is beneficial to human health. With a growing older adult population, a better understanding of older adult’s use of public transit and physical activity among public transit users is needed. We aim to describe public transit use among older adults from 2015 to 2022 within a nationally representative sample. Using the 2015-2022 National Health and Aging Trends Study (n = 38,970), we examined the prevalence of public transit use among adults aged 72 years and older. We found that 8.0% of older adults use public transit to get to places outside of their home. However, the prevalence of public transit use varied by gender, age group, and year of observation. Women (6.3%) used public transit less than men (8.5%), and public transit use prevalence was lower at older ages (ages 72-74, 7.8%; ages 90+, 3.6%. Public transit use was consistent across years 2015 to 2019 (8.5%), before it decreased drastically in 2020 (3.3%) and remained low in 2021 (5.2%) and 2022 (4.7%). In 2021 and 2022 public transit users averaged 48.8 more daily minutes of physical activity than non-users. These results suggest demographic and temporal trends in public transit use among older adults. Given that older adults report 7-10 non-driving years at the end-of-life, public transit could be an attractive option for this growing population. The pandemic reshaped the transportation landscape, and many public transit systems have not recovered pre-pandemic ridership. Future efforts should integrate age friendly policies to increase older adult ridership.

